# Parathyroid hormone stimulates bone regeneration in an atrophic non-union model in aged mice

**DOI:** 10.1186/s12967-023-04661-y

**Published:** 2023-11-23

**Authors:** Maximilian M. Menger, Anne L. Tobias, David Bauer, Michelle Bleimehl, Claudia Scheuer, Michael D. Menger, Tina Histing, Matthias W. Laschke

**Affiliations:** 1 Department of Trauma and Reconstructive Surgery, Eberhard Karls University Tuebingen, BG Trauma Center Tuebingen, 72076 Tuebingen, Germany; 2 Institute for Clinical and Experimental Surgery, Saarland University, 66421 Homburg/Saar, Germany

**Keywords:** Non-union, Parathyroid hormone, Segmental defect, Bone regeneration, Fracture healing, Angiogenesis, Mice, Aging, Inflammation

## Abstract

**Background:**

Non-union formation still represents a major burden in trauma and orthopedic surgery. Moreover, aged patients are at an increased risk for bone healing failure. Parathyroid hormone (PTH) has been shown to accelerate fracture healing in young adult animals. However, there is no information whether PTH also stimulates bone regeneration in atrophic non-unions in the aged. Therefore, the aim of the present study was to analyze the effect of PTH on bone regeneration in an atrophic non-union model in aged CD-1 mice.

**Methods:**

After creation of a 1.8 mm segmental defect, mice femora were stabilized by pin-clip fixation. The animals were treated daily with either 200 μg/kg body weight PTH 1–34 (n = 17) or saline (control; n = 17) subcutaneously. Bone regeneration was analyzed by means of X-ray, biomechanics, micro-computed tomography (µCT) imaging as well as histological, immunohistochemical and Western blot analyses.

**Results:**

In PTH-treated animals bone formation was markedly improved when compared to controls. This was associated with an increased bending stiffness as well as a higher number of tartrate-resistant acid phosphatase (TRAP)-positive osteoclasts and CD31-positive microvessels within the callus tissue. Furthermore, PTH-treated aged animals showed a decreased inflammatory response, characterized by a lower number of MPO-positive granulocytes and CD68-positive macrophages within the bone defects when compared to controls. Additional Western blot analyses demonstrated a significantly higher expression of cyclooxygenase (COX)-2 and phosphoinositide 3-kinase (PI3K) in PTH-treated mice.

**Conclusion:**

Taken together, these findings indicate that PTH is an effective pharmacological compound for the treatment of non-union formation in aged animals.

## Introduction

Despite increasing insights into the molecular and cellular mechanisms of fracture repair, non-union formation remains a major complication in orthopedic and trauma surgery [1]. In clinical practice, the treatment of non-unions is highly challenging and requires extensive revision surgery. Thus, non-union formation is not only associated with significant pain, loss of function and prolonged rehabilitation, but causes also a substantial economic burden for the health care system [2].

Due to the fact that the aged population is steadily increasing worldwide, the treatment of geriatric patients has become one of the major challenges in clinical practice [3]. The process of bone repair in the elderly is subject to physiological alterations, including decreased differentiation and proliferation of stem cells [4, 5] as well as delayed chondrogenesis and osteochondral ossification [6]. Accordingly, numerous clinical studies have reported that the aged population is at an increased risk for delayed fracture healing and non-union formation [7, 8]. To overcome this problem, there is a substantial need for the development of effective treatment strategies.

Parathyroid hormone (PTH), a peptide hormone secreted by the parathyroid glands, is a key regulator of calcium hemostasis in the body [9]. PTH raises the serum calcium levels by stimulating the reabsorption of calcium in the kidney and by stimulating osteoclast differentiation and proliferation, leading to osteoclastic bone resorption and calcium release from bone [10]. Moreover, PTH is the only clinically approved drug for the treatment of osteoporosis in the elderly. Of interest, several experimental studies demonstrated that PTH accelerates fracture healing [11–13] and stimulates bone regeneration in bone defects [14]. However, it is not known whether PTH also promotes the healing of atrophic non-unions in the aged.

To clarify this we analyzed in the present study, the effects of PTH treatment on atrophic non-unions using a well-established and reliable non-union model in aged CD-1 mice [15]. Bone regeneration was analyzed by means of X-ray, biomechanics, micro-computed tomography (µCT) imaging as well as histological, immunohistochemical and Western blot analyses.

## Materials and methods

### Animals

A total number of 34 male and female CD-1 mice with a body weight of 35–45 g and an age of 18–20 months were used. The age of 18–20 months was chosen according to reports of others, demonstrating age-associated physiological alterations and tumor development after 16–18 months in male and 18 months in female CD-1 mice [16]. The animals were bred at the Institute for Clinical and Experimental Surgery, Saarland University, Germany, and housed at a regular light and dark cycle with free access to tap water and standard pellet food (Altromin, Lage, Germany).

All experiments were performed according to the German legislation on the protection of animals and the National Institutes of Health (NIH) Guide for the Care and Use of Laboratory Animals (Institute of Laboratory Animal Resources, National Research Council, Washington DC, USA). The experiments were approved by the local governmental animal protection committee (permit number: 04/2019).

### Surgical procedure

Mice were anesthetized by intraperitoneal (i.p.) injection of ketamine (75 mg/kg body weight, Ursotamin®, Serumwerke Bernburg, Bernburg, Germany) and xylazine (15 mg/kg body weight, Rompun®, Bayer, Leverkusen, Germany). The pin-clip model using a segmental defect served as control and was performed as described previously [15]. Under aseptic conditions, a ~ 4 mm medial parapatellar incision was created at the right knee and the patella was dislocated laterally. After drilling a hole (diameter of 0.50 mm) into the intracondylar notch, a distally flattened pressfit 24 Gauge needle (diameter of 0.55 mm) was implanted intramedullary and the wound was closed. The pin was flattened at the distal end to avoid secondary dislocation. After insertion of the pin, the diaphysis of the femur was exposed by a lateral approach. Subsequently, a custom-made clip of 6 mm length was implanted ventrodorsally into the femur and lateral of the already implanted pin. A gap size of 1.8 mm was created by means of a spherical trephine under permanent saline solution cooling. Moreover, the periosteum was stripped 2 mm proximally and distally of the gap along the longitudinal axis of the femoral bone. The implant position was confirmed by radiography (MX-20, Faxitron X-ray Corporation, Wheelin, IL, USA). All procedures were done under an operating microscope, guaranteeing a high level of precision. For analgesia the mice received tramadol-hydrochloride (Grünenthal, Aachen, Germany) in the drinking water (1 mg/mL) 1 day prior to surgery until 3 days after surgery.

### Experimental protocol

Seventeen mice were daily treated with 200 μg/kg body weight PTH 1–34 (Bachem AG, Budendorf, Switzerland) dissolved in 100 µL saline, subcutaneously (PTH group). Control animals (n = 17) received an equal amount of saline (control group), subcutaneously. The used PTH dosage corresponds to other experimental studies investigating the effects of PTH on fracture healing in mice [13]. At 2 weeks [n = 5 each group (3 male; 2 female)] and 10 weeks [n = 9 each group (5 male; 4 female)] the animals were euthanized by an overdose of anesthetics and the femora were excised for further µCT and histological analyses. Additional animals were euthanized accordingly at 2 weeks [n = 3 each group (2 male; 1 female)] and tissue was harvested for Western blot analyses.

### X-ray analysis

At 2 and 10 weeks after surgery the animals were anesthetized and lateral radiographs of the osteotomized femora were performed. Bone healing was analyzed according to the Goldberg score with stage 0 indicating radiological non-union, stage 1 indicating possible union and stage 2 indicating radiological union [17].

### µCT analysis

The specimens were scanned (Skyscan 1176, Bruker, Billerica, MA) at a spatial resolution of 9 μm with a standardized setup (tube voltage: 50 kV; current: 200 µA; intervals: 0.4°; exposure time: 3500 ms; filter: 0.5 mm aluminum). Images were stored in three-dimensional arrays. To express gray values as mineral content (bone mineral density; BMD), calcium hydroxyapatite (CaHA) phantom rods with known BMD values (0.250 and 0.750 g CaHA/cm^3^) were employed for calibration. The region of interest (ROI) defining the novel bone was contoured manually excluding any original cortical bone. The thresholding allowed the differentiation between poorly and highly mineralized bone. The threshold to distinguish between poorly and highly mineralized bone was based upon visual inspection of the images, qualitative comparison with histological sections and other studies investigating bone repair and callus tissue by µCT [18, 19]. A BMD with more than 0.642 g/cm^3^, resulting in gray values of 98–255, was defined as highly mineralized bone. Poorly mineralized bone was assumed to have a BMD value between 0.410 g/cm^3^ and 0.642 g/cm^3^, resulting in gray values of 68–97.

The following parameters were calculated from the callus region of interest for each specimen: poorly mineralized bone volume (PM), highly mineralized bone volume (HM), bone volume fraction of tissue volume (BV/TV), bone surface (BS) density (BS/TV), trabecular thickness, trabecular separation and trabecular number.

### Biomechanical analysis

After removal of the soft tissue and the implants, the bending stiffness of the isolated femora was measured by a 3-point-bending device using a non-destructive approach. This allowed the subsequent use of the specimens for µCT as well as histological and immunohistochemical analyses and, thus, a reduction of the number of laboratory animals. Due to the different stages of healing, the loads, which had to be applied, markedly varied between individual animals. Loading was stopped individually in every case when the actual load-displacement curve deviated more than 1% from linearity. Bending stiffness (N/mm) was calculated from the linear elastic part of the load-displacement diagram [20].

### Histology and histomorphometry

After biomechanical testing and µCT analysis, bones were fixed in paraformaldehyde for 24 h. Subsequently, the specimens were embedded in a 30% sucrose solution for another 24 h and then frozen at − 80° C. Longitudinal sections through the femoral axis with a thickness of 4 μm were cut by the Kawamotos film method [21, 22] for histomorphometric analyses and stained with Safranin-O. At a magnification of 12.5× (Olympus BX60 Microscope, Olympus, Shinjuku, Japan; Zeiss Axio Cam and Axio Vision 3.1, Zeiss) structural indices were calculated according to the recommendations of Gerstenfeld et al. [23]. The following histomorphometric parameters of the bone defects were evaluated: (i) total callus area, (ii) bone callus area, (iii) cartilaginous callus area and (iv) fibrous callus area. The total callus area was defined as the entire osseous, cartilaginous and fibrous callus tissue between the two drilling holes of the clip outside of the cortices. Pre-existing cortical bone of the proximal and distal fragment, however, was excluded. Each area was marked and calculated using the ImageJ analysis system (NIH, Bethesda, USA).

In addition, tartrate-resistant acid phosphate (TRAP) activity was analyzed in the callus tissue at 2 and 10 weeks after surgery. For this purpose, longitudinal sections of 4 μm were incubated in a mixture of 5 mg naphotol AS-MX phosphate and 11 mg fast red TR salt in 10 mL 0.2 M sodium acetate buffer (pH 5.0) for 1 h at 37 °C. Sections were counterstained with methyl green and covered with glycerin gelatin. TRAP-positive multinucleated cells (three or more nuclei each cell) were counted. In the specimens, one high-power field (HPF, 400× magnification) was placed in a standardized manner in the central region of the callus, while three additional HPFs were placed on each site of the periosteal callus.

### Immunohistochemistry

To analyze the cellular composition within the callus tissue of atrophic non-unions at 2 and 10 weeks after surgery, longitudinal sections with a thickness of 4 μm were cut. For the immunohistochemical detection of microvessels, sections were stained with a monoclonal rat anti-mouse antibody against the endothelial cell marker CD31 (1:100; Abcam, Cambridge, UK). A goat anti-rat IgG-Alexa555 antibody served as secondary antibody (1:100; Life Technology, Eugene, USA). Cell nuclei were stained with Hoechst 33342 (2 µg/mL; Sigma-Aldrich, Taufkirchen Germany). To detect the neutrophilic granulocyte marker myeloperoxidase (MPO) and the macrophage marker CD68, sections were stained with a polyclonal rabbit anti-mouse antibody against MPO (1:100; Abcam) and a polyclonal rabbit anti-mouse antibody against CD68 (1:100; Abcam). A goat anti-rabbit IgG-antibody (1:200; Dianova, Hamburg, Germany) served as corresponding secondary antibody.

In the specimens, the number of CD31-positive microvessels as well as MPO- and CD68-positive cells was counted. For this purpose, one HPF was placed in a standardized manner in the central region of the callus, while three additional HPFs were placed on each site of the periosteal callus.

### Western blot analysis

Protein expression within the callus tissue was determined by Western blot analysis, including the expression of vascular endothelial growth factor (VEGF), cyclooxygenase (COX)-2 and phosphoinositide 3-kinase (PI3K). The callus tissue was frozen and stored at − 80 °C until required. Analyses were performed from callus tissue at 2 weeks after surgery (n = 3 each group). After saving the whole protein fraction, analysis was performed using the following antibodies: rabbit anti-mouse VEGF (1:300, Abcam, Cambridge, UK), COX-2 (1:30, Abcam) and mouse anti-mouse PI3K (1:100, Santa Cruz Biotechnology, Heidelberg, Germany). Primary antibodies were followed by corresponding horseradish peroxidase-conjugated secondary antibodies (1:1000, R&D Systems). Protein expression was visualized by means of luminol-enhanced chemiluminescence after exposure of the membrane to the Intas ECL Chemocam Imager (Intas Science Imaging Instrument GmbH, Göttingen, Germany) and normalized to β-actin signals (1:1000, mouse anti-mouse β-actin, Santa Cruz Biotechnology) to correct for unequal loading.

### Statistical analysis

All data are given as means ± SEM. After testing the data for normal distribution (Kolmogorov–Smirnov test) and equal variance (*F*-test), comparisons between the two groups were performed by an unpaired Student’s *t*‐test. For non‐parametrical data, a Mann–Whitney *U*‐test was used. All statistics were performed using the SigmaPlot 13.0 software (Jandel Corporation, San Rafael, CA, USA). A p‐value of < 0.05 was considered to indicate significant differences.

## Results

### X-ray analysis

**Fig. 1:**
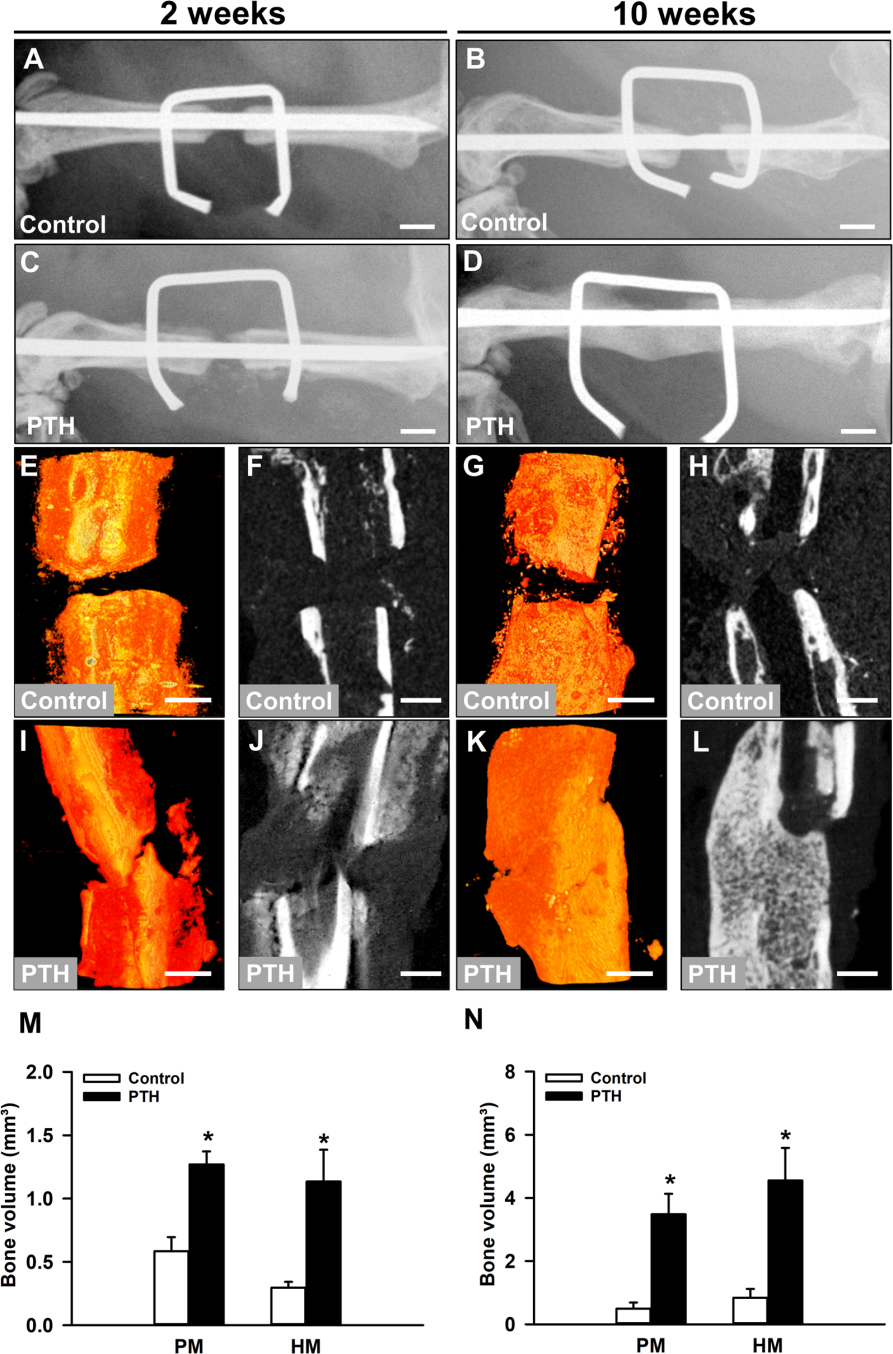
**A**–**D** Representative X-rays of femora of controls (**A**, **B**) and PTH-treated mice (**C**, **D**) at 2 (**A**, **C**) and 10 weeks (**B**, **D**) after surgery. Scale bars: 1 mm. Representative µCT-3D reconstructions (**E**, **G**, **I**, **K**) and transversal µCT images (**F**, **H**, **J**, **L**) of controls (**E**–**H**) and PTH-treated animals (**I**–**L**) at 2 (**E**, **F**, **I**, **J**) and 10 weeks (**G**, **H**, **K**, **L**) after surgery. Scale bars: 0.5 mm. **M**, **N** Poorly (PM) and highly mineralized (HM) bone volume of the callus tissue of controls (white bars, n = 5 at 2 weeks after surgery, n = 9 at 10 weeks after surgery) and PTH-treated mice (black bars, n = 5 at 2 weeks after surgery, n = 9 at 10 weeks after surgery) at 2 (**M**) and 10 weeks (**N**) after surgery, as assessed by µCT analyses. Mean ± SEM; *p < 0.05 vs. control

The radiographic analysis demonstrated a complete lack of osseous bridging in the control group throughout the entire observation period (Fig. 1A, B). X-rays showed a reliable non-union formation in all control mice with a large persisting gap between the adjoining rounded bone fragments (Fig. 1B). However, in PTH-treated animals the X-rays already indicated first signs of callus formation at 2 weeks after surgery (Fig. 1C) and even osseous bridging in 6 out of 9 (67.7%) animals at 10 weeks after surgery (Fig. 1D). Accordingly, the radiographic analysis revealed a significantly higher Goldberg score in PTH-treated animals at 10 weeks after surgery when compared to controls (0.0 ± 0.0 vs. 1.1 ± 0.3; *p < 0.05 vs. control).

### µCT analysis

In line with our X-ray analysis, µCT imaging showed a lack of osseous bridging in control animals throughout the entire 10-weeks observation period (Fig. 1E–H). In contrast, in the PTH group we observed signs of callus formation already at 2 weeks after surgery (Fig. 1I, J) and successful osseous bridging at 10 weeks after surgery (Fig. 1K, L), indicating an improved bone regeneration. Accordingly, the µCT analysis demonstrated a significantly higher amount of poorly and highly mineralized bone tissue in PTH-treated animals at 2 and 10 weeks after surgery (Fig. 1M, N).

**Fig. 2:**
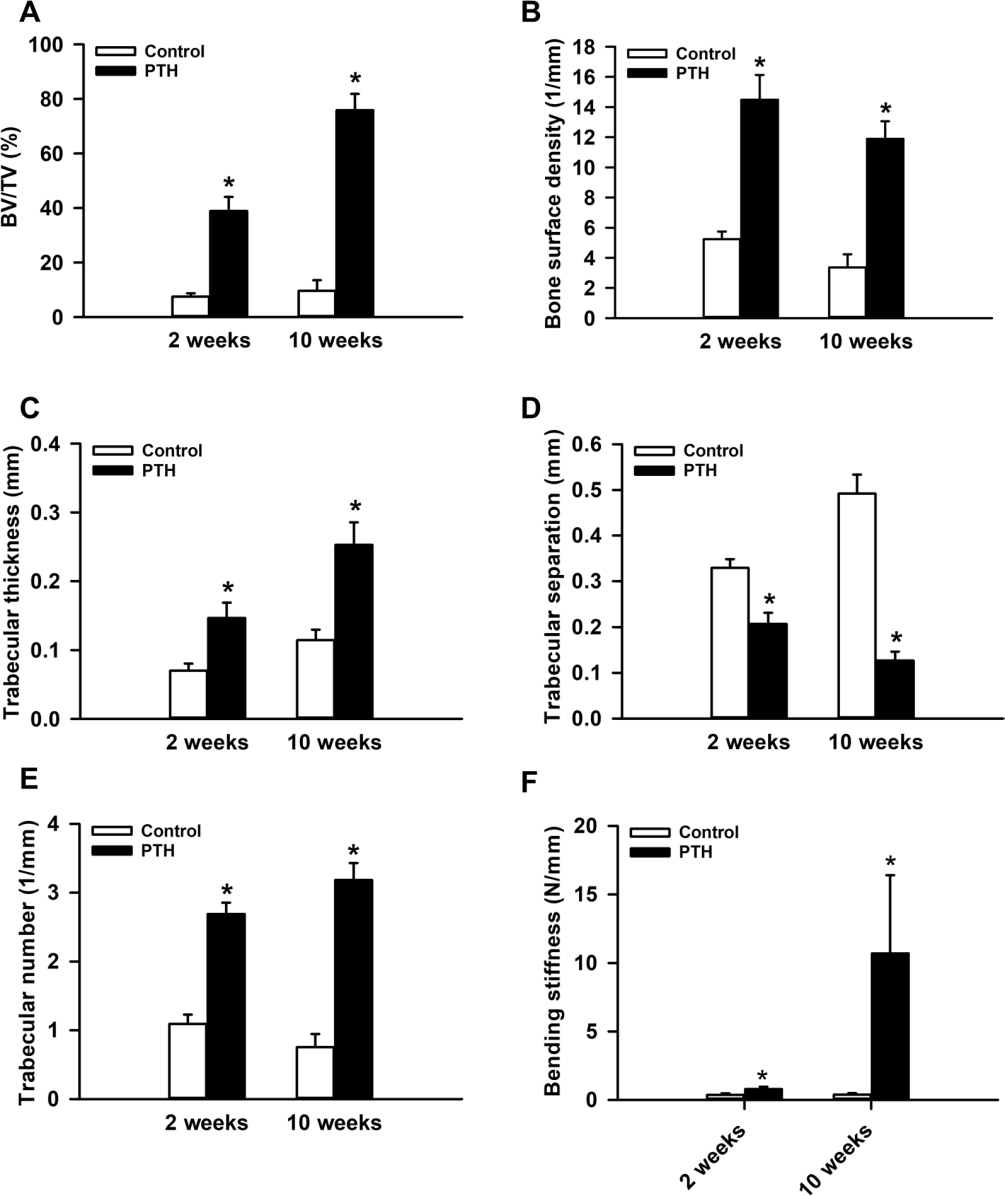
**A**–**E** BV/TV (%) (**A**), bone surface density (1/mm) (**B**), trabecular thickness (mm) (**C**), trabecular separation (mm) (**D**) and trabecular number (1/mm) (**E**) of the callus tissue of controls (white bars, n = 5 at 2 weeks after surgery, n = 9 at 10 weeks after surgery) and PTH-treated mice (black bars, n = 5 at 2 weeks after surgery, n = 9 at 10 weeks after surgery) at 2 and 10 weeks after surgery, as assessed by µCT analyses. **F** Bending stiffness (N/mm) of femora of controls (white bars, n = 5 at 2 weeks after surgery, n = 9 at 10 weeks after surgery) and PTH-treated mice (black bars, n = 5 at 2 weeks after surgery, n = 9 at 10 weeks after surgery) at 2 and 10 weeks after surgery, as assessed by biomechanical analysis. Mean ± SEM; *p < 0.05 vs. control

In addition, we found a significantly higher BV/TV and bone surface density in PTH-treated mice at 2 and 10 weeks after surgery when compared to controls (Fig. 2A, B). Further analyses of the trabecular architecture demonstrated a significantly higher trabecular thickness in the PTH group at 2 and 10 weeks after surgery (Fig. 2C). This was associated with a significantly lower trabecular separation and, accordingly, a significantly higher trabecular number in PTH-treated animals when compared to controls throughout the 10-weeks observation period (Fig. 2D, E).

### Biomechanical analysis

Femora of PTH-treated mice exhibited a significantly higher bending stiffness at 2 and 10 weeks after surgery when compared to controls (Fig. 2F). Notably, the bending stiffness of femora of control animals remained < 1 N/mm, indicating a complete failure of fracture healing (Fig. 2F).

### Histomorphometric and immunohistochemical analysis

**Fig. 3:**
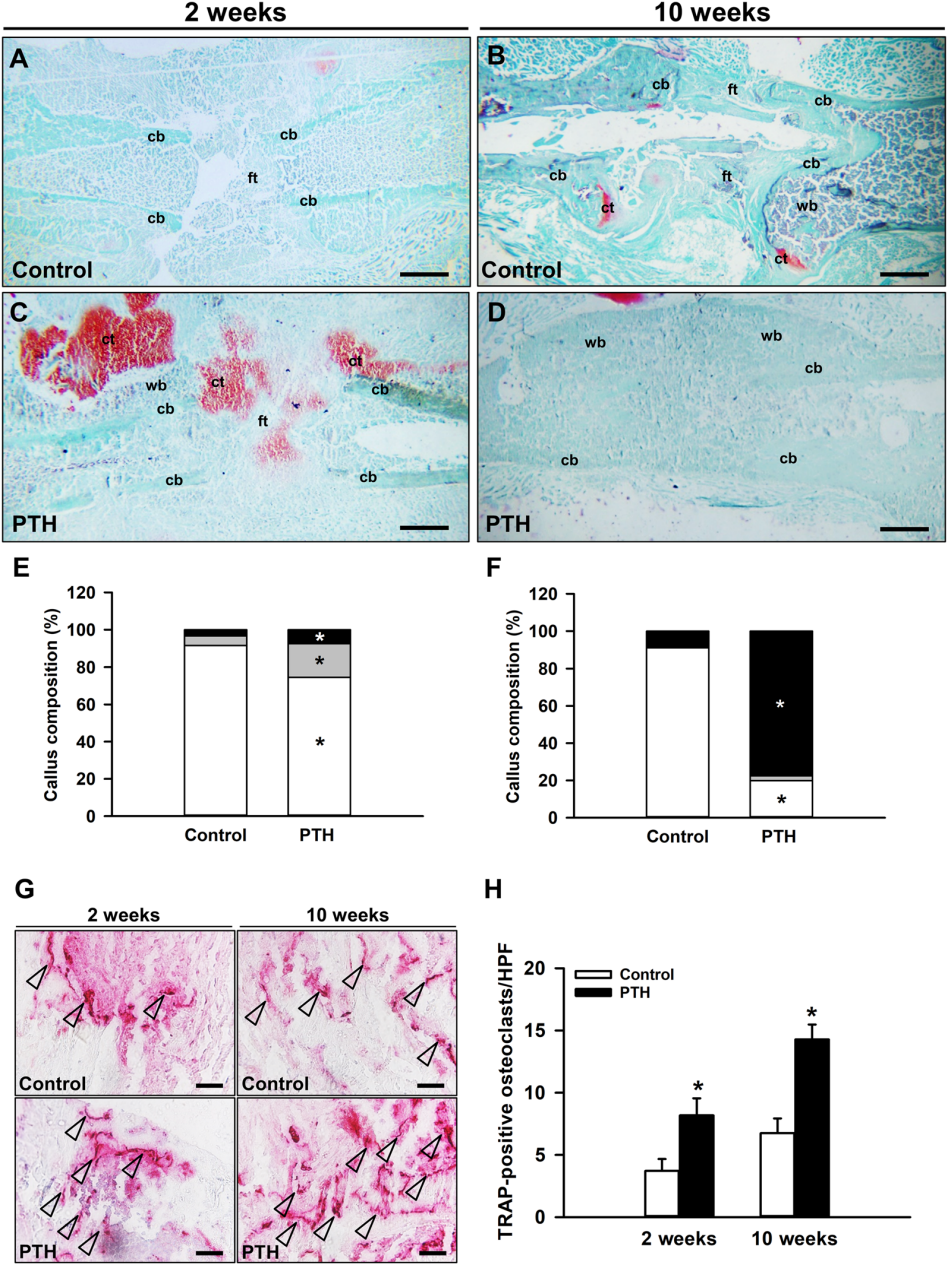
**A**–**D** Representative histological images of Safranin-O-stained femora of controls (**A**, **B**) and PTH-treated mice (**C**, **D**) at 2 (**A**, **C**) and 10 weeks (**B**, **D**) after surgery. Fibrous tissue (ft), cartilaginous tissue (ct), woven bone (wb) and cortical bone (cb) are indicated. Scale bars: 0.5 mm. **E**, **F** Callus composition (%), including fibrous tissue (white), cartilaginous tissue (gray) and osseous tissue (black), of the callus of controls (n = 5 at 2 weeks after surgery, n = 9 at 10 weeks after surgery) and PTH-treated mice (n = 5 at 2 weeks after surgery, n = 9 at 10 weeks after surgery) at 2 (**E**) and 10 (**F**) weeks after surgery, as assessed by histomorphometric analysis. **G** Representative histological images of TRAP-positive osteoclasts (arrowheads) within the callus tissue of controls and PTH-treated mice at 2 and 10 weeks after surgery. Scale bars: 25 μm. **H** TRAP-positive osteoclasts/HPF within the callus tissue of controls (white bars, n = 5 at 2 weeks after surgery, n = 9 at 10 weeks after surgery) and PTH-treated mice (black bars, n = 5 at 2 weeks after surgery, n = 9 at 10 weeks after surgery) at 2 and 10 weeks after surgery, as assessed by histological analysis. Mean ± SEM; *p < 0.05 vs. control

Histomorphometric analysis demonstrated a complete lack of osseous bridging in the control group at 2 and 10 weeks after surgery with abundant fibrous tissue between the bone fragments (Fig. 3A, B). In PTH-treated animals, distinct signs of endochondral ossification could be detected at 2 weeks after surgery (Fig. 3C), resulting in osseous bridging at 10 weeks (Fig. 3D). This indicates successful bone regeneration. Quantitative analyses revealed a significantly higher fraction of bone and cartilaginous tissue at 2 weeks after surgery in the PTH group when compared to controls, whereas the fraction of fibrous tissue was significantly lower (Fig. 3E). At 10 weeks, the callus tissue in the control group still consisted mainly of fibrous tissue, whereas in PTH-treated animals it mainly consisted of newly formed bone tissue. Notably, the amount of cartilaginous tissue did not significantly differ between the two study groups at 10 weeks after surgery (Fig. 3F).

**Fig. 4:**
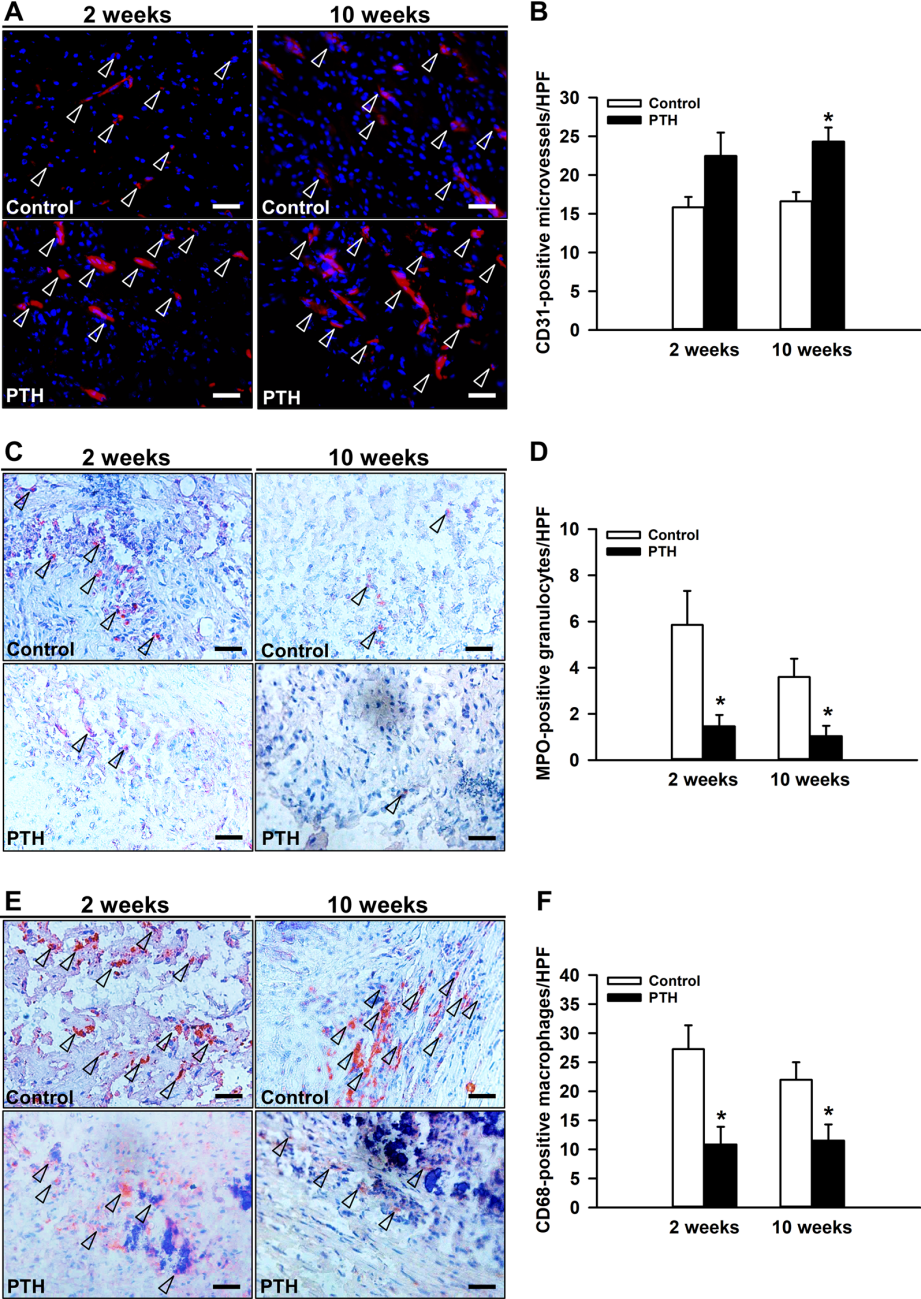
**A** Representative immunohistochemical images of CD31-positive microvessels (arrowheads) within the callus tissue of controls and PTH-treated mice at 2 and 10 weeks after surgery. Scale bars: 25 μm. **B** CD31-positive microvessels/HPF within the callus tissue of controls (white bars, n = 5 at 2 weeks after surgery, n = 9 at 10 weeks after surgery) and PTH-treated mice (black bars, n = 5 at 2 weeks after surgery, n = 9 at 10 weeks after surgery) at 2 and 10 weeks after surgery, as assessed by immunohistochemical analysis. Mean ± SEM; *p < 0.05 vs. control. **C** Representative immunohistochemical images of MPO-positive cells (arrowheads) within the callus tissue of controls and PTH-treated mice at 2 and 10 weeks after surgery. Scale bars: 25 μm. **D** MPO-positive cells/HPF within the callus tissue of controls (white bars, n = 5 at 2 weeks after surgery, n = 9 at 10 weeks after surgery) and PTH-treated mice (black bars, n = 5 at 2 weeks after surgery, n = 9 at 10 weeks after surgery) at 2 and 10 weeks after surgery, as assessed by immunohistochemical analysis. Mean ± SEM; *p < 0.05 vs. control. **E** Representative immunohistochemical images of CD68-positive cells (arrowheads) within the callus tissue of controls and PTH-treated mice at 2 and 10 weeks after surgery. Scale bars: 25 μm. **F** CD68-positive cells/HPF within the callus tissue of controls (white bars, n = 5 at 2 weeks after surgery, n = 9 at 10 weeks after surgery) and PTH-treated mice (black bars, n = 5 at 2 weeks after surgery, n = 9 at 10 weeks after surgery) at 2 and 10 weeks after surgery, as assessed by immunohistochemical analysis. Mean ± SEM; *p < 0.05 vs. control

In addition, the analysis of the number of TRAP-positive osteoclasts within the callus tissue, demonstrated a significantly higher number of positive cells in PTH-treated animals at 2 and 10 weeks when compared to controls (Fig. 3G, H). Moreover, the analysis of the vascularization of the callus tissue by immunohistochemical staining of CD31-positive microvessels, demonstrated a slightly higher number of microvessels at 2 weeks after surgery, and a significantly higher number at 10 weeks in the PTH-group when compared to controls (Fig. 4A, B). Furthermore, the analysis of the inflammatory response in the callus tissue by staining MPO-positive granulocytes and CD68-positive macrophages revealed in PTH-treated animals a significantly lower number of MPO-positive granulocytes and CD68-positive macrophages at 2 and 10 weeks after surgery (Fig. 4C–F).

### Western blot analysis

**Fig. 5:**
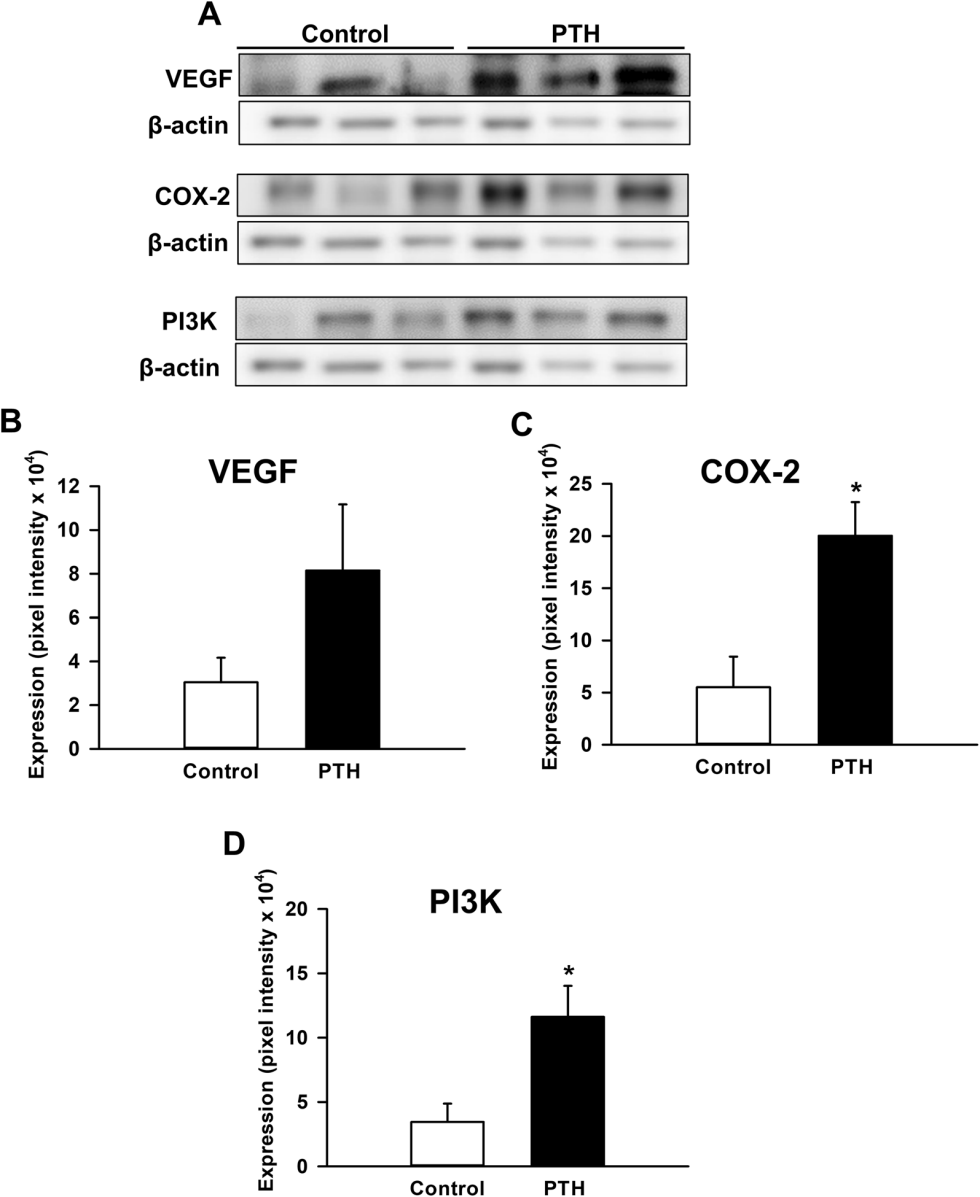
**A** Representative Western blots of VEGF, COX-2, PI3K and β-actin expression within the callus tissue of controls and PTH-treated mice at 2 weeks after surgery. **B**–**D** Expression of VEGF (pixel intensity × 10^4^) (**B**), COX-2 (pixel intensity × 10^4^) (**C**) and PI3K (pixel intensity × 10^4^) (**D**) within the callus tissue of controls (white bars, n = 3) and PTH-treated mice (black bars, n = 3) at 2 weeks after surgery, as assessed by Western blot analysis. Mean ± SEM; *p < 0.05 vs. control

Western blot analyses of the callus tissue at 2 weeks after surgery revealed an over twofold higher expression of VEGF in the PTH group when compared to controls. However, this difference did not prove to be statistically significant (Fig. 5A, B). In contrast, the expression of COX2 and PI3K was significantly higher within the callus tissue of PTH-treated mice when compared to controls (Fig. 5A, C, D).

## Discussion

Non-union formation still represents a major complication in trauma and orthopedic surgery, especially in geriatric patients. Therefore, the aim of the present study was to clarify, whether this problem may be overcome by stimulating bone regeneration with PTH. The major findings of the study are that in a murine atrophic non-union model, PTH treatment improves bone formation, resulting in successful osseous bridging at 10 weeks after surgery. This was associated with a higher bending stiffness and an increased vascularization within the callus tissue of femora of PTH-treated animals when compared to controls.

In clinical practice there are variabilities on the definition of delayed healing and non-union formation [24]. Non-unions are defined by the U.S. Federal Drug Administration council as ‘failure to achieve union by 9 months since the injury, and for which there has been no signs of healing for 3 months’ [25]. However, others define non-union formation in long bones after a period of 6 months without radiological signs of fracture healing [25]. In general, it should be considered that the diagnosis of non-union always includes both the radiological and clinical examination of the patient [26].

In the present study, we used the non-union model by Garcia et al. [27], which we recently established in geriatric CD-1 mice [15]. Notably, aging affects the process of bone regeneration by multiple factors, including (i) a decreased differentiation and proliferation of stem cells, (ii) a delay in chondrogenesis and osteochondral ossification as well as (iii) a dysfunction in the bone vascular system [15, 28]. In a recent study, we showed that aged CD-1 mice exhibit a delayed process of callus remodeling and, thus, a delayed fracture healing when compared to young adult animals. Interestingly, the overall healing capacity is not affected by aging, as indicated by the finding that both aged and young adult animals achieve complete femoral bone healing 4 to 5 weeks after fracture [29]. Non-union formation in these animals has been considered as lack of healing for at least 10 weeks [15, 27, 30]. This in line with human femoral fractures, which require around 12 weeks for normal healing and non-union is defined as lack of healing after 24 weeks, thus double of the normal healing period [27]. Our results demonstrate that PTH treatment leads to osseous bridging at 10 weeks after surgery and, therefore, overcomes failed fracture-healing and non-union formation.

As a species on the lower phylogenetic scale, mice possess a great potential for bone repair. Hence, the development of a reliable and reproducible non-union model is highly challenging. Garcia et al. [27] established such a model by creating a critical size femoral defect and performing additional periosteal stripping with subsequent fixation by a ‘pin-clip’ device. Interestingly, in this model a reliable non-union formation was only achieved with additional periosteal stripping after creation of the 1.8 mm femoral defect, whereas animals with intact periosteum demonstrated a partial healing at 10 weeks after surgery [27]. The periosteum is a highly vascularized tissue, which plays a vital role in the process of physiological bone regeneration by providing the cortical blood supply [31] and serves as a reservoir for osteoprogenitor cells [32]. Moreover, it is well known that periosteal stripping impairs fracture healing [33, 34]. These findings may explain the additional deterioration of bone repair after periosteal stripping in the present non-union model.

Fracture healing is characterized by the formation of a soft callus tissue, which gradually transforms into bone by the resorption of calcified cartilage and the formation of novel bone tissue [35, 36]. Interestingly, our histomorphometric analysis showed a significantly higher fraction of cartilaginous tissue in PTH-treated aged animals at 2 weeks after surgery as a clear sign of endochondral ossification. This resulted in the formation of novel bone tissue and osseous bridging at 10 weeks after surgery. In contrast, no signs of endochondral ossification were evident in control animals throughout the 10-weeks observation period. Moreover, the callus tissue of PTH-treated animals showed a significantly higher number of TRAP-positive osteoclasts. Osteoclasts are vital for cartilage resorption and callus remodeling, promoting the formation of mature novel bone tissue [37]. Therefore, it may be assumed that PTH treatment stimulates bone regeneration in aged mice by inducing endochondral ossification and osteoclast-mediated callus remodeling.

Angiogenesis plays a crucial role for fracture repair. In fact, newly formed blood vessels allow the delivery of nutrients to the fracture site and the infiltration of cells that are essential for callus remodeling [38]. The importance of vascularization is also highlighted by several experimental studies demonstrating that the pharmacological inhibition of angiogenesis by TNP-470, nonsteroidal anti-inflammatory drugs or fumagillin impairs fracture repair and eventually leads to non-union formation [39–41]. Interestingly, our immunohistochemical analysis showed an increased number of CD31-positive microvessels within the callus tissue of PTH-treated animals. This was associated with an over twofold higher expression of VEGF in the PTH group when compared to controls. VEGF is recognized as a main growth factor for the stimulation of angiogenesis [42]. Moreover, several studies indicated that VEGF is directly involved in the process of bone regeneration [43, 44]. Accordingly, inhibition of VEGF expression has been shown to impair fracture repair and to result in non-union formation [45], whereas stimulation of VEGF expression can accelerate fracture healing [46]. Notably, the effects of VEGF on fracture repair exceed the sole stimulation of angiogenesis, but also involve the direct stimulation of endochondral and intramembranous fracture healing as well as stem cell recruitment [43, 44]. Therefore, it may be speculated that PTH improves bone regeneration in aged mice not only by the stimulation of vascularization but also by directly inducing novel bone formation.

The inflammatory phase is a crucial part of successful bone regeneration, as neutrophilic granulocytes and macrophages remove injured and devitalized tissue within the callus and mesenchymal stem cells are directed to the fracture site [37, 47]. A dysregulated, perturbated or even chronic inflammation, however, can impair the process of bone regeneration and even lead to healing failure [48, 49]. Aged individuals exhibit higher levels of circulating pro-inflammatory cytokines, resulting in a chronic inflammatory status, referred to as “inflamm-aging” [50]. Accordingly, we previously detected an increased number of pro-inflammatory macrophages within the callus tissue of non-unions in aged mice when compared to young animals [51]. Moreover, in the present study our immunohistochemical analysis demonstrated that the improved bone regeneration in PTH-treated animals is associated with a significantly lowered number of neutrophilic granulocytes and macrophages within the callus tissue. In line with these findings, Clark et al. [28] recently showed that the inhibition of macrophage recruitment results in improved fracture healing with increased bone volume in aged mice. Thus, PTH treatment most likely inhibits the overshooting inflammatory response in non-unions of aged mice by reducing the number of granulocytes and macrophages within the callus, leading to the observed improved bone regeneration.

Cyclooxygenases (COXs) are necessary for the generation of prostaglandins, prostacyclins and thromboxanes during inflammation. Notably, COX-2 is highly expressed in chondrocytes and chondroprogenitors during the early stage of bone repair [52]. Moreover, Naik et al. [53] observed a delayed bone remodeling in aged mice with a decreased COX-2 expression during the early inflammatory phase of bone repair, highlighting the crucial role of COX-2 in bone healing. In the present study, Western blot analyses demonstrated during the early healing period of 2 weeks a significantly increased expression of COX-2 in PTH-treated mice. Hence, these findings indicate that PTH-treatment improves bone regeneration in aged mice by upregulating COX-2 expression within the callus tissue during the early stage of healing.

PI3K signaling is a pathway activated through receptor tyrosine kinases (RTKs) and G-protein coupled receptors, playing a vital role in cell metabolism and proliferation as well as the regulation of gene expression [54]. Moreover, there is evidence that PI3K signaling is directly involved in the regulation of osteoblastogenesis and skeletal remodeling by controlling osteoblast proliferation and differentiation [55]. Furthermore, Scanlon et al. [56] recently reported that knock-in mice with a global increase in PI3K signaling (gCbl^YF^) exhibit an improved femoral bone healing, which is characterized by an enhanced proliferation of periosteal cells during the first days of fracture repair. In the present study, we found a significantly increased expression of PI3K within the callus tissue of aged PTH-treated mice. Therefore, it may be speculated that the improved bone healing observed in PTH-treated animals is mediated in part also by the activation of PI3K signaling.

In summary, our results demonstrate that PTH treatment significantly improves the regeneration of atrophic non-unions in aged mice. This was associated with an increased number of osteoclasts and CD31-positive microvessels as well as an enhanced expression of COX-2 and PI3K within the callus tissue of PTH-treated animals during the early healing phase. As PTH is a clinically approved drug [13], it may be a promising candidate for the future treatment of non-union formation in aged patients.

## Data Availability

The datasets during and/or analyzed during the current study available from the corresponding author on reasonable request.
