# Correction to: Parathyroid hormone stimulates bone regeneration in an atrophic non-union model in aged mice

**DOI:** 10.1186/s12967-024-05594-w

**Published:** 2024-09-09

**Authors:** Maximilian M. Menger, Anne L. Tobias, David Bauer, Michelle Bleimehl, Claudia Scheuer, Michael D. Menger, Tina Histing, Matthias W. Laschke

**Affiliations:** 1 Department of Trauma and Reconstructive Surgery, Eberhard Karls University Tuebingen, BG Trauma Center Tuebingen, Tuebingen, 72076 Germany; 2 Institute for Clinical and Experimental Surgery, Saarland University, Homburg/Saar, 66421 Germany

Following publication of the original article [1], we have been notified that there is a spelling mistake in the abstract and body text of the article.

It is now:

200 mg/kg

It should be:

200 µg/kg

The original article was updated.
